# Creating Memories for False Autobiographical Events in Childhood: A Systematic Review

**DOI:** 10.1002/acp.3220

**Published:** 2016-04-08

**Authors:** Chris R. Brewin, Bernice Andrews

**Affiliations:** ^1^ University College LondonLondonUK; ^2^ Royal Holloway, University of LondonLondonUK

## Abstract

Using a framework that distinguishes autobiographical belief, recollective experience, and confidence in memory, we review three major paradigms used to suggest false childhood events to adults: imagination inflation, false feedback and memory implantation. Imagination inflation and false feedback studies increase the belief that a suggested event occurred by a small amount such that events are still thought unlikely to have happened. In memory implantation studies, some recollective experience for the suggested events is induced on average in 47% of participants, but only in 15% are these experiences likely to be rated as full memories. We conclude that susceptibility to false memories of childhood events appears more limited than has been suggested. The data emphasise the complex judgements involved in distinguishing real from imaginary recollections and caution against accepting investigator‐based ratings as necessarily corresponding to participants' self‐reports. Recommendations are made for presenting the results of these studies in courtroom settings. © 2016 The Authors Applied Cognitive Psychology Published by John Wiley & Sons Ltd.

The early 1990s saw a well‐documented concern that some psychotherapy patients might be wrongly convinced by their therapists that they had experienced sexual abuse in childhood and recover corresponding memories of events that had not occurred (Lindsay & Read, 1994; Loftus, 1993). The possibility was raised that a variety of suggestive therapeutic techniques could create illusory memories of abuse having happened. Subsequently, experiments were designed that showed false memories of childhood events could be created under laboratory conditions. Although the success of these experiments has been widely accepted, summarising their results accurately is complex because different paradigms and memory measures have been employed, and the findings have been very variable. This may explain why to date there have been no systematic reviews of the research on creating false childhood memories in adults despite undoubted professional and public interest in the topic, as illustrated by a recent special issue of *Applied Cognitive Psychology* titled ‘Reshaping memories through conversations: Considering the influence of others on historical memories of abuse’. The two aims of this review are to develop a framework for describing the nature of these laboratory‐produced memories and to use this framework to document more precisely what these studies have and have not shown. The review should be of benefit to researchers, experts testifying in legal settings and psychotherapists attempting to evaluate the status of childhood memories in a therapeutic context.

Three experimental paradigms have been most commonly employed to mimic possible therapy‐inspired distortions to childhood autobiographical memory (Laney & Loftus, 2013; Schacter & Loftus, 2013). In order of the amount of explicit suggestion and apparent corroboration provided to participants, these are the following: imagination inflation studies, in which participants are instructed to repeatedly imagine events that have not occurred; ‘false feedback’ studies, in which participants are given mainly generic false information suggesting they were likely to have experienced an event and memory implantation studies in which the suggested occurrence of the false event is supported by false testimony from an individual's family members or individually doctored photographs. Although the latter two paradigms overlap somewhat, we treat them separately here because they typically differ both in the procedures used and the outcome data (changes in self‐ratings versus investigator‐based ratings). The majority of studies using all three of these paradigms imply the practical relevance of their work by including mention of memory for child abuse or trauma, therapy or legal proceedings.

Some initial reactions to the publication of these studies were to conclude that imagining events that had not happened can ‘change memory’ (Garry & Polaschek, 2000) or that it was easy to implant false childhood memories (Wade, Garry, Read, & Lindsay, 2002). More recently, articles have suggested that, on average, false childhood memories can be implanted in almost 40% of participants (Strange, Wade, & Hayne, 2008). A State of the Science Editorial (Laney & Loftus, 2013) concluded that ‘all these techniques produced false memories in substantial numbers of research participants’ and went on to describe further research that has been ‘key to establishing the reliability of laboratory‐induced false memory, as well as their relevance to the precipitating issue of recovered memories of child sexual abuse’ (p. 139). Other authors (Conway, 2013) have concluded that ‘wholly false memories are more common than previously thought, especially for childhood events and, even more alarmingly, it turns out to be almost trivially easy to create false memories in others’ (p. 567).

The findings of this research have also been widely accepted within psychology textbooks, with statements (D. A. Bernstein, 2014) such as: ‘Merely thinking about certain objects or events or hearing sounds or seeing photos associated with them appears to make false memories of them more likely’ (p. 223); or (Nolen‐Hoeksema, Fredrickson, Loftus, & Lutz, 2014) ‘memories of entirely fictional events have been shown to be implantable under controlled conditions…it is possible to induce such memories by merely having people imagine fictional renderings of their pasts’ (pp. 289–290). In the absence of a systematic review, however, it is unclear whether these statements accurately capture the available data.

One reason for caution is that a number of researchers in the field have observed how studies vary considerably in their definition and operationalisation of false memory (Scoboria, Mazzoni, Kirsch, & Relyea, 2004; Smeets, Merckelbach, Horselenberg, & Jelicic, 2005). Moreover, discriminating whether one's memories are true or false is a complex process involving high‐level evaluative procedures (Johnson, 1997; Lindsay & Johnson, 2000). Perhaps as a consequence, the results of studies using all three paradigms have been highly variable. These observations suggest the value of applying an explicit framework to the existing data in order to evaluate the literature. In this article, we describe such a framework grounded in the previous work of autobiographical memory researchers and apply it to the results of the three major methods of suggesting false childhood memories.

## A Framework for Discriminating Truth and Falsity in One's Own Autobiographical Memories

The term ‘autobiographical memory’ refers to the totality of what can be recalled about the self and includes knowledge of both facts and events. In thinking about event memory, a useful starting point is the ‘remember‐know’ distinction (Mandler, 1980; Tulving, 1985) that has been so influential in experimental studies of episodic memory retrieval: Participants can experience their past knowledge of a stimulus event either as a feeling of familiarity (‘knowing’ they have seen it before without retrieving the context) or as a full recollective experience accompanied by sensory images arising from the original context (‘remembering’). With autobiographical memory, individuals can similarly know or believe on the basis of external evidence that an event has occurred (such that they visited New York when aged 2 years) without necessarily having any corresponding recollective experience such as visual images.

Unlike memory for laboratory stimuli, however, for which feelings of familiarity provide the main source of ‘know’ judgments, the belief that an autobiographical event has occurred may rest on a wide variety of additional types of plausibility information either known to the individuals concerned or supplied by others (e.g. pictures of the family visit to New York). Such information is important to evaluate the plausibility of specific recollective experiences. Just as it is possible to know or believe something occurred without recollection, it is possible to have recollection without belief: An example is when a recollective experience such as a visual image is thought to be inaccurate or completely false (Mazzoni, Scoboria, & Harvey, 2010; Scoboria et al., 2014). Observations of such non‐believed recollective experiences have featured from the start in studies of false memory implantation (Hyman, Husband, & Billings, 1995).

Even if the event referred to in the recollective experience is plausible, the elements of that experience may still be imaginary rather than real. Research on source memory has considered in detail the problems of establishing the truth of one's own recollective experiences and emphasised the complexity of the evaluative processes involved (Johnson, 2006). Johnson suggested that a variety of factors are involved, including the amount of perceptual and contextual detail available, the plausibility of what is recalled, the existence of supporting knowledge and beliefs, information about mental operations such as imagination that might provide an alternative explanation and the decision criteria adopted. Based on existing findings, she proposed that false memories are more likely when procedures are used that increase the similarity between true and imagined events, such as those that increase the amount of imagery available, that decrease the accessibility of disconfirming information and that lead to features of real events being imported.

Given these complexities in discriminating whether experiences correspond to true or false events, it has been necessary to stipulate criteria by which researchers can conclude that individuals have fully recollected a particular episode from their past. A prominent view from autobiographical memory research (Brewer, 1986) is that three elements are needed: the belief that the episode was personally experienced by the self, the presence of (usually visual) imagery and the confidence that this imagery is a veridical record of the originally experienced episode. Similar criteria have been employed by other leading researchers. Pezdek and colleagues suggested that recollective experiences are only likely to be accepted if the events are initially judged as plausible and consistent with script‐relevant knowledge (Pezdek, Finger, & Hodge, 1997). Hyman additionally proposed that the creation of false memories involves the acceptance of the false event as plausible, the construction of an image and narrative of the event and the source memory error of confusing the constructed memory as a personal recollection (Hyman, Gilstrap, Decker, & Wilkinson, 1998). A related model (Mazzoni, Loftus, & Kirsch, 2001) distinguished between a perception that an autobiographical event is plausible, the belief that it occurred to them and a corresponding recollective experience that is interpreted as a memory.

Empirical research supports the argument that it is important to distinguish between these different kinds of judgement. For example, telling people that certain events, such as receiving an enema, were highly prevalent during their childhood produces greater belief in the possibility of having experienced those events, but not actual memories of the experience (Hart & Schooler, 2006; Pezdek, Blandon‐Gitlin, Lam, Hart & Schooler, 2006). Similarly, in false feedback studies, increases in the belief that an event has occurred have been noted even in participants who later say they are positive that the event did not happen (D. M. Bernstein, Laney, Morris, & Loftus, 2005b). Other research has found that whereas perceptual, re‐experiencing and emotional features of an event predict recollective experience but not a belief that the event actually occurred, event plausibility strongly predicts a belief that the event occurred but only weakly predicts recollective experience (Scoboria et al., 2014).

Some authors of studies using the three main childhood false memory paradigms explicitly refer to both a belief in an event having occurred and recollective experiences as ‘memories’ (Laney, Fowler, Nelson, Bernstein, & Loftus, 2008) and Scoboria et al. (2014) note that it is often far from easy to distinguish between different types of memory judgement. A problem with studies using investigator ratings of memory accounts is that memory terms are sometimes not understood or used correctly by those taking part in memory studies, and their responses can be affected by ambiguous wording (Smeets et al., 2006). For example, Smeets and colleagues (Smeets, Telgen, Ost, Jelicic, & Merckelbach, 2009) found that two‐thirds of their participants appeared to endorse having ‘memories’ of non‐existent footage of the assassination of the Dutch politician Pim Fortuyn, but more detailed questioning determined that in 80% of cases, their statements referred to beliefs rather than recollective experiences. Thus, while to outside observers, there may appear to be a ‘memory’ based on the language used, subjectively the individual may believe the event occurred without having an accompanying recollective experience (Ost, Granhag, Udell, & Roos af Hjelmsäter, 2008; Otgaar, Scoboria, & Smeets, 2013). Even if there is a recollective experience, this may not be believed (Mazzoni et al., 2010; Scoboria et al., 2014).

Synthesising this previous body of work, it appears that there is considerable support for the utility of Brewer's (1986) framework distinguishing three types of memory judgement: (1) a belief that the event occurred (termed here ‘autobiographical belief’); (2) a corresponding recollective experience; and (3) confidence in the veracity of that memory experience (termed here ‘memory confidence’). While autobiographical belief is a component of memory, we suggest that a ‘full’ memory for an event, whether true or false, should ideally rest on the combination of the second and third elements. We refer to recollective experiences that have not been demonstrated to be held with confidence as ‘partial’ memories.

These three types of memory judgement are readily discerned in the literature. Table 1 presents some of the most widely used self‐report measures and investigator‐based (false) memory definitions that have been employed in imagination inflation, false feedback and memory implantation studies. As previously remarked by several authors, most imagination inflation and false feedback studies have primarily assessed autobiographical belief and have had participants rate their confidence that an event has occurred, typically on an 8‐point scale anchored with ‘definitely did not happen’ at one end and ‘definitely did happen’ at the other. Several researchers (Mazzoni et al., 2001; Smeets et al., 2005) have noted, further, that increases in confidence cannot be assumed to correspond to a belief that an event has occurred if confidence ratings remain in the lower half of the scale (i.e. closer to ‘did not happen’ than to ‘did happen’). Therefore, in summarising the literature, it is important to describe not only the scales that are used but also the mean ratings before and after any intervention.

**Table 1:** Main measures and definitions corresponding to autobiographical belief, recollective experience and memory confidence in studies of false memory for childhood events

Measure	Paradigm	Memory aspect assessed	Description
Life Events Inventory (Garry et al., 1996)	Imagination inflation	Autobiographical belief	Self‐report rating: 1 ‘definitely did not happen’ to 8 ‘definitely did happen’
Autobiographical Beliefs and Memory Questionnaire (Scoboria et al., 2004)	Imagination inflation, false feedback, memory implantation	Autobiographical belief	Self‐report rating: 1 ‘definitely did not happen’ to 8 ‘definitely happened’
		Recollective experience	
			Self‐report rating: 1 ‘no memory at all’ to 8 ‘clear and complete memory’
Food History Inventory (Bernstein et al., 2005a)	False feedback	Autobiographical belief	Self‐report rating: 1 ‘definitely did not happen’ to 8 ‘definitely did happen’
Memory/belief form (Bernstein et al., 2005a)	False feedback	Autobiographical belief versus recollective experience	Self‐report rating: 1 ‘a specific memory for the event’; 2 ‘a belief that the event happened, but [without] a specific memory’; 3 ‘positive that the event did not happen to you’
Memory definition (Loftus & Pickrell, 1995)	Memory implantation	Recollective experience	Investigator‐based rating: 1 full recall; 2 partial recall including remembering parts of event and speculations about how and when it might have happened
Memory definition (Hyman et al., 1995, Experiment 2)	Memory implantation	Recollective experience	Investigator‐based rating: Memory included as false recall if descriptions included some of the false information or elaborations consistent with it – had to actually describe false event – some ‘saw’ or believed but did not describe (very clear: incorporated more of false information and often elaborated; less clear: incorporated less critical or no false information but elaborated in a way only possible given false information)
Memory definition (Hyman & Pentland, 1996)	Memory implantation	Recollective experience, memory confidence	Investigator‐based rating: Memory rating (clear = reports of target event, consistent elaborations and statements that event was a memory; partial = consistent elaborations with some statements of remembering, but none of actual target event; no memory but trying to recover = described an image or related self‐knowledge but no clear claim to remember event; no memory = none of the above)
Memory rating (Hyman & Pentland, 1996)	Memory implantation	Memory confidence	Self‐report rating: 1 ‘not confident’ to 7 ‘very confident’
Memory definition (Pezdek et al., 1997)	Memory implantation	Recollective experience	Investigator‐based rating: An event was operationally defined as remembered if the subject recalled specific details of the event that were not included in the description read
Memory definition (Porter et al., 1999)	Memory implantation	Recollective experience, memory confidence, autobiographical belief	Investigator‐based rating: complete = report of remembering event, agreed with and/incorporated the information clues into the report and reported additional information; partial = recalled information or imagery pertaining to the event, but not recalled in its entirety or uncertainty whether memory was real
Memory definition (Lindsay et al., 2004)	Memory implantation	Recollective experience	Investigator‐based rating: memory = S appeared to believe he or she was remembering the suggested event; partial memory = S described images associated with suggested event but did not appear to experience those images as memories of the event per se
Memory rating (Strange et al., 2008)	Memory implantation	Autobiographical belief versus recollective experience	Self‐report rating: As I think about the event, I can actually remember it rather than just know that it happened (1 ‘not at all’ to 7 ‘as much as any memory’)

The self‐report measures used in the imagination inflation and false feedback studies to assess recollective experience generally ask participants to rank their experience on a scale from ‘no memory at all’ to a ‘clear and complete memory’ or to contrast a ‘memory’ with a ‘belief’ that an event happened. These methods rely on participants distinguishing beliefs and recollective experiences, but do not explicitly require participants to describe images or recount narratives, or to consider whether such images are necessarily veridical. Particularly when ‘memories’ are simply being contrasted with ‘beliefs’ in a dichotomous rating, little information is available about the extent of the memory or of the individual's confidence in it.

The memory implantation studies have, in contrast, primarily relied on investigator‐based ratings and have gone to considerable lengths to characterise the nature of participants' response. From the beginning, investigators recognised that many participants were uncertain about their recollective experiences and attempted to distinguish ‘partial’ memories (e.g. memories that were vague, incomplete or not necessarily believed to be real) from ‘full’ or ‘complete’ memories (Hyman et al., 1995; Loftus & Pickrell, 1995). This has been difficult because of the need to separate participants' memory of what they were asked to imagine by the experimenters, often over repeated sessions, from their depiction of what they believed actually happened to them. For this reason, as shown in Table 1, several investigators have required participants to elaborate or to produce information additional to what was suggested. However, this additional material also needs to be accepted as true in relation to the suggested event (i.e. ‘memory confidence’ also needs to be assessed).

One of the most stringent formulations has been that of Hyman and Pentland (1996), who defined ‘clear’ memories as reports of a core aspect of the target event with ‘consistent elaborations and statements that the event was a memory’. Reading the published examples produced by this method (e.g. Hyman & Billings, 1998), however, illustrates that even in the case of ‘clear’ memories, participants are sometimes unconfident about their experiences or incorporate elements from their own biography that do not always fit the suggested scenarios. Other investigators have equally recognised that a visual image may not always be classified as an actual memory but have sometimes been less specific about requiring evidence of confidence in memory when defining a ‘full’ recollection, for example, by using as a criterion ‘if the subject appeared to believe he or she was remembering the suggested event’ (Lindsay, Hagen, Read, Wade, & Garry, 2004) (p. 151).

Although many individual false memory studies discuss the distinctions between autobiographical belief, recollective experience or memory confidence in relation to their data, these constructs have not been used as a frame of reference for organising and summarising the results of the field as a whole. In the sections that follow, we review the evidence for change in memory for childhood events brought about by each of the three primary false memory paradigms. In seeking to quantify this, we follow the literature in describing the proportion of participants who respond to suggestions and the amount and nature of mean changes on the memory scales employed. Because it is these raw, unadjusted data that best capture the degree of change achieved, a meta‐analytic approach that converted the data into standardised effect sizes would be inappropriate: Instead, we have calculated weighted means that preserve the original metrics. This analytic approach also allows for the fact that the memory implantation studies do not consistently report appropriate contrast conditions from which an effect size could be calculated: Although participants are usually required to recall true as well as false events, when the data are reported, there are generally differences in the numbers of events, the procedures used or the way they are coded, which prevents more general comparison. Meta‐analysis of the imagination inflation and false feedback studies would in any case be of limited value because of the insufficiently frequent inclusion of theoretically relevant moderator variables. As summarised in the tables, the vast majority of the 61 studies included in this review were conducted with young adults in mainly college student populations (only four included older adults): Consideration of false memories in children is beyond the scope of the paper.

## Imagination Inflation

Vividly imagining large numbers of words, sentences, actions, and so on can lead to confusion over whether specific words have been spoken or specific actions performed or whether this only happened in the person's imagination. The act of imagining such actions can lead to an increase in the belief that they actually happened (Anderson, 1984; Foley, Johnson, & Raye, 1983). Researchers applying similar procedures to childhood memories (Garry, Manning, Loftus, & Sherman, 1996) coined the term ‘imagination inflation’. In the studies to be reviewed, participants typically complete a checklist of distinctive events that might have happened in childhood (such as getting stuck in a tree or putting one's hand through a window), rating them on an autobiographical belief/event confidence scale. Events (usually those rated as unlikely to have occurred) are then assigned either to an ‘imagine’ or a control condition and subsequently re‐rated for likelihood of having occurred. The experimenter typically guides the participants' imagination with specific instructions to focus on different attributes of the ‘memory’, augmented with the requirement subsequently to answer questions about the event as if it had happened, to write in detail about the hypothetical event or both.

### Method

A search was conducted on Web of Science, Current Contents and MEDLINE using the topic search term ‘imagination inflation’. To be included, a study had to contain distinct conditions measuring increases in the belief that a childhood autobiographical event had happened after an imagination manipulation and in a non‐imagination (control) condition, report sufficient data to allow these conditions to be contrasted and measure autobiographical belief both before and after the manipulation. Of the 156 potential articles found up to the end of 2015, 12 met the inclusion criteria. Additional hand‐searching of cited and citing references produced a further four qualifying articles, resulting in a total of 16 articles incorporating 19 different datasets. Data were extracted independently and cross‐checked by both authors.

The studies are summarised in Table 2. In the third column, this details any additional relevant within‐subjects and between‐subjects conditions that featured in the studies over and above the test of the imagination inflation effect. The remaining columns present the results. In the literature, tests of the imagination inflation effect are reported in a number of different ways. Early studies typically reported the percentage of all events that received increased belief/confidence ratings after the intervention, regardless of the magnitude of the increase, divided by whether they were imagined or not imagined. This method, which collapses datapoints across events and participants, gives an indication numerically of whether any imagination inflation has occurred. However, unlike within‐subjects designs in which the participant remains the unit of analysis, the non‐independence of observations compromises the application of conventional statistical tests. Where such data are reported, they are tabulated in the fourth column of Table 2, along with any restrictions (e.g. percentages only calculated on events initially rated as unlikely to have occurred). The specific events imagined are not described as there were often multiple events, and these were not necessarily the same for all participants.

**Table 2:** Imagination inflation studies of childhood events

Authors	Participants	Additional relevant conditions	% of events with increased belief ratings^a^	Significant inflation effect	Mean belief/recollection increase on 8‐point scale^a^	Mean increase beyond scale midpoint^a^
Garry et al. (1996)	38 college students		(pre‐test ratings 1–4) 34% imagined 25% not imagined	Mean ratings not tested		
Paddock, Joseph et al. (1998) Study 1	98 college students		(pre‐test ratings 1–4) 38% imagined^b^ 21% not imagined^b^	Yes		
Paddock, Joseph et al. (1998) Study 2	106 middle aged adults			No		
Paddock et al. (1999)	94 college students		(pre‐test ratings 1–4) 47% imagined 35% not imagined	Yes		
Heaps and Nash (1999)	55 college students			Yes (1‐tailed test only)	*Belief*	No
					0.44 imagined	
					0.18 not imagined	
Clancy et al. (1999)	12 women with recovered memories	Recovered memory status B/S	(pre‐test ratings 1–4) range: 25–19% imagined	Mean ratings not tested		
	12 controls		12% not imagined^b^			
Horselenberg et al. (2000) Study 1	34 college students			No	*Belief* (1–7)	No
					0.37 imagined	
					0.27 not imagined	
Horselenberg et al. (2000) Study 2	45 college and high school students			Yes	*Belief* (1–7)	No
					0.51 imagined	
					−0.02 not imagined	
Pezdek and Eddy (2001)	43 college students, 32 older adults	Young versus old B/S	(pre‐test ratings 1–4) 39% imagined	No	*Belief* (pretest ratings 1–4)	No
			25% not imagined		1.13 imagined	
					0.68 not imagined	
Mazzoni and Memon (2003)	72 college students	Improbable (skin) versus probable (tooth) event B/S; imagined versus exposed versus not imagined W/S		Yes	*Belief* range:	Probable event:
					0.86–0.42 imagined	
						N/A^c^ Improbable event: No
					−0.30 to −1.34 exposed/not imagined	
Sharman et al. (2004)	67 college students	Imagined versus paraphrasing W/S; 0, 1, 3 or 5 repetitions W/S		No	*Belief* b All < 1.00	No
Pezdek et al. (2006)	145 college students	High versus low plausible events W/S	range: 38–21% imagined 28–23% not imagined	Yes (high plausible event only)	*Belief* range: 0.94–0.21 imagined	No
					0.39–0.22 not imagined	
Sharman and Barnier (2008)	78 college students	Positive versus negative events W/S		Yes: *Belief and Recollection*	(pre‐test ratings 1–4)	Yes: *Belief* No: *Recollection*
					*Belief* range:	
					3.64–3.52 imagined	
					2.22–1.90 not imagined	
					*Recollection* range: 3.09–2.90 imagined	
					1.50–1.13 not imagined	
Sharman and Scoboria (2009)	60 college students	Belief analysis: moderate versus low event plausibility W/S; Recollection analysis: high versus moderate versus low event plausibility W/S		Yes: *Belief and Recollection*	*Belief* range: (pretest ratings 1–4)	Moderate and low plausibility events:
					0.67–0.40 imagined	
						No: *Belief*
						No: *Recollection*
					0.18–0.16 not imagined	
						High plausibility events: N/A^c^
					*Recollection* range:	
					(pre‐test ratings 1–8)	
					1.14–0.08 imagined	
					0.61 to −0.04 not imagined	
von Glahn et al. (2012) Study 2	60 college students	Number of imagined details generated (0,3,6) W/S		Yes	*Belief* range: 0.30–0.21^b^ imagined	No
					−0.04^b^ not imagined	
Bays et al. (2012)	135 college students	Number of imaginings (0,1,5) W/S; High versus low plausibility events W/S		No: *Belief and Recollection*	*Belief* range: 0.13 to −0.08 imagined	No
					−0.18 to −0.33 not imagined	
					*Recollection* range: 0.34 to 0.04 imagined	
					−0.10 to −0.18 not imagined	
Bays, Foley, and Zabrucky (2013)	151 college students	Number of imaginings (0, 1, 5) W/S		No	*Belief* range: (pretest ratings 1–4)	
					1.26–1.05 imagined	
					0.69 not imagined	
Marsh et al. (2014) Study 1	47 college students	Imagined from first versus third person perspective W/S		Yes (third person perspective only)	*Belief* range: 1.11–0.47 imagined	No
					0.04 not imagined	
Marsh et al. (2014) Study 2	64 college students	As for Study 1		Yes (third person perspective only)	*Belief* range: 0.64–0.20 imagined	N/A^c^
					−0.17 not imagined	

Cells left blank where relevant data not reported.

B/S = between‐subjects; W/S = within‐subjects.

a Belief (confidence) and recollection ratings in listed studies are all on 1‐ to 8‐point scales unless otherwise specified; where ranges are given, means and percentages vary according to condition; scales include the following: Life Events Inventory (LEI: Garry et al., 1996) with authors' modified events; Autobiographical Beliefs and Memory Questionnaire (ABMQ: Scoboria et al., 2004).

b Estimated from published figures.

c N/A = not applicable as all mean pre‐test ratings are above or near midpoint.

This way of presenting the results is silent about the amount of any increase and where this occurs on the belief/confidence scale, impeding quantification and interpretation of the effects (Horselenberg et al., 2000; Pezdek & Eddy, 2001). One solution has been to compare the means of the same items when they were imagined and not imagined (i.e. treating items as cases). The consequent non‐independence of observations once again compromises the application of conventional statistical tests. Reliable estimates of the presence of a significant imagination inflation effect can however be obtained from tests based on mean scores for individual participants, providing that the tests' assumptions are not violated (Garry et al., 1996). The next column of Table 2 therefore reports, where available, the results of such between‐group statistical tests. These include comparisons of post‐intervention group means adjusted for pre‐intervention means, comparisons of group change scores and tests of time by group interactions in repeated‐measures ANOVAs. The final two columns quantify the size of this effect in terms of the mean increase on the 8‐point scale and whether this increase takes the average score above the scale midpoint. It was not possible to quantify the effect by reporting the number of participants whose scores moved from below to above the midpoint as these data were rarely reported.

### Results and discussion

The studies are relatively homogeneous in that, with some exceptions, participants imagine events initially rated as unlikely that occurred to them before the age of 10 years. Visual inspection of the percentages of all memories with increased belief ratings after events that had been either imagined or not imagined (Table 2, column 4) indicates a very consistent advantage for the imagined condition, even though it was only a minority of participants who changed their ratings (Garry, Sharman, Wade, Hunt, & Smith, 2001). Consistent with these data are the changes in mean ratings for the imagined and control conditions shown in column 6 of Table 2, where these are available. Visual inspection confirms that in the great majority of cases, positive mean change is higher after imagination versus control conditions.

Seventeen out of the 19 datasets reported valid tests of the imagination inflation effect for a belief that the event had happened based on individuals' mean scores. As shown in Table 2, 11 of these found a statistically significant difference in the belief as a result of imagining versus not imagining and six did not. In two datasets (Sharman & Barnier, 2008; Sharman & Scoboria, 2009), there was additionally an imagination inflation effect for recollective experience measured by means of a memory scale, but the number of participants with clear complete memories was not reported in either study. A similar effect was not found in the only other study assessing memory (Bays, Zabrucky, & Gagne, 2012). Of the 14 datasets reporting means pre‐intervention and post‐intervention, 13 found that the increase attributable to imagination over the non‐imagination control condition was equivalent to 1 point or less on the 8‐point scale (or on a 7‐point scale in the Horselenberg et al. studies) and that mean scores remained in the lower half of the scale. In one study reporting such data, Garry et al. (1996) noted that large jumps to eight on the post‐test confidence scale occurred for six of the 112 imagined items and two of the 119 not‐imagined items.

In one exception to this general pattern of results (Sharman & Barnier, 2008), mean belief ratings for imagined negatively and positively valenced childhood events jumped from 1.86 to 5.50 and 2.02 to 5.54, respectively. Increases were also larger than average in the control condition, with ratings rising from 1.82 to 3.72, and from 1.97 to 4.19, for negatively and positively valenced events, respectively.

The data indicate fairly conclusively that on average the imagination inflation paradigm increases participants' beliefs that events, originally perceived as unlikely to have happened, are more likely to have occurred than they first estimated. The procedure influences a minority of participants, and to date, the effects appear to be small in magnitude. Post‐test ratings tended to fall below the scale midpoint, and it has been suggested that this effect can be more accurately characterised as reducing the belief that the event did not happen rather than increasing the belief that the event did happen (Mazzoni et al., 2001; Smeets et al., 2005). From a source memory perspective (Johnson, 1997, 2006), acceptance of the suggested event is likely to be increased by the additional available imagery, but the effect may be limited by participants' awareness of the mental operations that preceded the eventual judgements.

An alternative explanation for the effect raised in the initial study by Garry et al. (1996) was that because most events are selected for having low confidence ratings initially, part of the effect may consist of regression to the mean. Consistent with this possibility, Pezdek and Eddy (2001) reported that whereas autobiographical belief increased during the course of the experiment for events initially rated as unlikely to have occurred, it decreased for events initially rated as likely to have occurred. The plausibility of this explanation can be assessed by inspecting the mean change in belief that occurred for control events also selected for being unlikely to have occurred. Regression effects should increase autobiographical belief for these events too. Table 2 indicates that in the nine datasets that reported a significant imagination inflation effect and gave the relevant group means, a change in autobiographical belief shown by the control group was positive in five cases and negative in four cases. This suggests that regression to the mean is unlikely to have made a substantial contribution to imagination inflation.

Another possibility discussed originally by Garry et al. (1996) is that imagination procedures can actually lead to the retrieval of true memories and that this explains at least some of the increase in subsequent confidence. In particular, the repeated opportunity to retrieve childhood events may result in hypermnesia (Erdelyi, 2010) such that true memories are spontaneously recovered (Hyman et al., 1995; Hyman & Pentland, 1996). In one of the few studies to have addressed this possibility, Mazzoni and Memon (2003) employed as events a medical procedure (having a skin sample taken from a little finger) that was known not to have been used in the country where the study was conducted and compared it with another procedure [having a milk (baby) tooth extracted] that was. This design ensured that any increase in confidence about the ‘impossible’ event occurring could not be attributed to a true memory. While confidence increased for both events, the magnitude of the increase obtained for the ‘impossible’ event was very small (less than half a scale point). Moreover, interpretation of statistical significance of their results was complicated by the combination of both events in the analysis and by the simultaneous decreased confidence in the control group who did not imagine the events.

Although the possibility that recall of true events might account for a proportion of the imagination inflation effect was not ruled out by the Mazzoni and Memon (2003) study, it seems unlikely to have been an important contributor to results using the original Life Events Inventory (LEI). This is because in that measure childhood events were quite specific, low‐frequency occurrences (e.g. ‘Broke a window with your hand’). This factor may, however, account for the exceptionally strong effects achieved by Sharman and Barnier (2008) who developed their own version of the LEI, replacing most of the original items with positive and negative events (Stefanie J. Sharman, personal communication 4 December 2014). Relative to the standard LEI, the description of the events presented as examples appears to have been less distinctive and to have included an element of judgement or interpretation (e.g. ‘you gave someone a gift for no special reason’). These features raise the possibility (i) that events were more easily overlooked when initially rated for belief in occurrence and (ii) that guided imagination was more likely to have prompted recall of a genuine past experience.

## False Feedback

A second group of studies has attempted to increase belief in childhood autobiographical events by providing false feedback to participants. In a typical design, participants complete a number of measures, including the confidence with which they believe certain events happened to them before the age of 10 (for example, that they liked or did not like certain foods), before being provided with false feedback and re‐rating their confidence in the event happening to them. The studies we review provide feedback ostensibly from a computer programme or psychotherapist, indicating that a particular event or preference was likely to have happened or applied to the participant at that age. The study may involve imagining the event or answering questions about it (i.e. using imagination inflation procedures) prior to the final re‐rating of confidence. A few of these studies also recorded the extent to which following feedback participants had some indication of an actual memory of the suggested event, rather than believing it had happened without being able to remember it.

A separate question of interest is the extent to which those who respond positively to the false feedback (typically called ‘Believers’ in these studies) differ from those who do not respond (‘Non‐believers’). ‘Believers’ are typically defined as responding to the intervention with at least a 1 point increase in confidence and reporting some belief or memory for the target event, although they are not required to report high levels of autobiographical belief and/or a recollective experience (D. M. Bernstein, Laney, Morris, & Loftus, 2005a; D. M. Bernstein et al., 2005b). As the primary focus of this review is to document the effects of false feedback versus no false feedback on autobiographical belief and recollective experience, means reported in Table 3 refer to the entire subsample exposed to the various conditions. However, secondary analyses concerning ‘Believers’ within qualifying studies are discussed in the text.

**Table 3:** False memory studies of uncorroborated childhood events (‘false feedback’ paradigm)

Authors	Participants	False childhood event (additional relevant B/S conditions)	Significant FF effect	Mean belief/recollection increase on 8‐point scale^a^	Mean increase beyond scale midpoint^a^	% memory or recollection post‐test
Mazzoni and Loftus (1998)	44 college students	Lost; abandoned; lonely and lost, all before age 3 years	Yes	*Belief* (collapsed across events) 2.26 FF; −0.16 no FF		
Mazzoni et al. (1999)	63 college students	Lost; bullied, both before age 3 years	Yes	*Belief* (collapsed across events) 1.15 FF; −0.47 no FF		
Mazzoni et al. (2001) Study 1	65 college students	Witnessed possession; almost choked. both before age 3 years (FF choked versus FF possession versus no FF)	Yes	*Belief* range: 1.68–1.54 FF 0.05 to −0.1 no FF	No	
Mazzoni et al. (2001) Study 2	71 college students	Witnessed possession; kidnapping threat (FF possession versus FF kidnap versus no FF)	Yes	*Belief* range: 1.48–0.87 FF 0.50–0.24 no FF	No	
Mazzoni et al. (2001) Study 3	57 college students	Witnessed possession (FF possession same culture versus FF other culture versus no FF)	Yes: same culture group only	*Belief* range: 0.84–0.15 FF −0.06 no FF	No	
Bernstein et al. (2005a) Study 1	131 college students	Ill after strawberry ice cream; sick after chocolate chip cookie (FF ill strawberry versus FF sick chocolate versus no FF)	No B/S test conducted	*Belief* range: 0.51 to −0.13 FF −0.23 to −0.36 no FF	No	No data for whole FF group and control group
Bernstein et al. (2005a) Study 2	204 college students	Ill after strawberry ice cream (FF + scenario versus FF + elaboration versus no FF)	No B/S test conducted	*Belief* range: 1.12–0.5 FF 0.16 no FF	No	No data for whole FF group and control group
Bernstein et al. (2005b) Study 2	180 college students	Sick after pickle or egg (feedback target: pickle versus egg)	Yes	*Belief* range: 0.88–0.63 FF 0.17–0.07 no FF	No	No data for whole FF group and control group
Scoboria et al. (2007) Study 1	56 college students	Bone density screening (FF prevalence and rationale versus FF prevalence versus no FF)	Yes: *Belief* FF Prevalence + rationale only No: *Recollection*	*Belief* range: 2.17–0.11 FF −0.69 no FF *Recollection* range: 0.11 to −0.12 FF −0.21 no FF	No: *Belief and Recollection*	
Scoboria et al. (2007) Study 2	156 college students	Bone density screening; skin sample taken (FF prevalence and rationale versus FF prevalence versus no FF)	Yes: *Belief* FF Prevalence + rationale only	*Belief* range: 1.61–0.87 FF −0.16 no FF	No: *Belief and Recollection*	
			No: *Recollection*			
				*Recollection* range:		
				0.14–0.07 FF		
				−0.09 no FF		
Laney, Morris et al. (2008) Study 1	97 college students	Loved to eat asparagus	Yes	*Belief* (pre‐test ratings 1–4) 2.60 FF; 0.20 no FF	No	*Recollection*: 22% FF 12% no FF *Belief*: 35% FF 28% no FF
Laney, Morris et al. (2008) Study 2	73 college students	Loved asparagus first time tried	Yes	*Belief* (pre‐test ratings 1–4) 2.50^b^ FF; 1.07^b^ no FF	No	*Recollection*: 28% FF 6% no FF *Belief*: 28% FF 38% no FF
Laney, Fowler et al. (2008)	320 college students	Loved (or hated) asparagus first time tried (loved versus hated asparagus)	No test of effect on whole FF group	*Belief* (1–7) range: (excludes Ps with both pre + post‐test ratings > 4) 2.21–1.39 FF 0.93 to −0.08 no FF	No: loved asparagus Yes: hated asparagus	*Recollection*: 17–7% FF 12–5% no FF *Belief*: 30–27% FF 49–41% no FF
Laney, Kaasa et al. (2008) Study 1	187 college students	Loved asparagus first time tried; sick after asparagus	Yes: Loved No: Sick	*Belief* (1–7) range: Loved^b^ 1.40 FF; −0.20 no FF Sick^b^ 0.70 FF; 0.30 no FF	No	
Berkowitz et al. (2008)	332 college students	Ear licked by Pluto (FF good Pluto versus FF bad Pluto versus no FF)	No B/S test conducted	*Belief* range: (pre‐test ratings 1–4) 1.12–0.36 FF 0.10 no FF	No	*Recollection*: range 3–2% FF *Belief*: range 37–27% FF No data for control group
Scoboria et al. (2008)	21 young adults	Sick on spoiled dairy	No	*Belief* (means not reported)		
Sharman and Calacouris (2010)	46 college students	Achievement event; affiliation event	Yes *Belief and Recollection*	(low pretest belief ratings) *Belief* range: 4.35–3.91 FF 2.76–1.80 no FF *Recollection* range: 3.87–3.28 FF 1.74–1.13 no FF	Yes: *Belief* (FF and No FF) Yes: *Recollection*	
Scoboria, Mazzoni, Jarry, & Bernstein (2012)	122 college students	Sick on peach yoghourt [personalised FF (PFF) versus no PFF, generalised FF (GFF) versus no GFF]	Yes: PFF only *Belief and Recollection*	(pretest belief rating 1–4) *Belief* range: 2.30–0.94 FF; 1.03 no FF	No: *Belief and Recollection*	(pretest belief rating 1–4) *Recollection*: 19% PFF 2% no PFF
				*Recollection* range: 1.70–0.13 FF; 0.21 no FF		*Belief*: 25% PFF 5% no PFF
Scoboria, Mazzoni, Jarry, & Shapero (2012) Study 1	42 college students	Sick on peach yoghourt	Yes: *Belief* (*Recollection* analysis on post‐test ratings only)	*Belief* 2.68 FF; 1.45 no FF *Recollection* 1.68 FF; 0.40 no FF	No *Belief and Recollection*	
Clifasefi et al. (2013)	125 college students	Sick after rum or vodka before age 16 years	Yes	*Belief* (alcohol drinkers only) 0.54 FF; −0.15 no FF	No	No data for whole FF group and control group

Cells left blank where relevant data not reported.

B/S = between‐subjects; FF = false feedback condition; No FF = no false feedback condition; P = Participants.

a Belief (confidence) and recollection ratings in listed studies are all on 1–8 point scales unless otherwise specified; where ranges are given, means and percentages vary according to condition; scales include the following: Life Events Inventory (LEI: Garry et al., 1996) with authors' modified events; Food History Inventory (FHI: Bernstein et al., 2005a); Autobiographical Beliefs and Memory Questionnaire (ABMQ: Scoboria et al., 2004); Food and Beverage History Questionnaire (FBHQ: Clifasefi et al., 2013).

b Estimated from published figures.

### Method

A search was conducted on Web of Science, Current Contents and MEDLINE using the joint topic search terms ‘false memor*’ and ‘childhood’. To be included, a study had to compare a false feedback condition in which individual participants were given specific information suggesting they personally were likely to have experienced the event with a no‐feedback control condition and measure autobiographical belief or recollective experience both before and after the feedback. The search produced 350 potential articles up to the end of 2015, and additional hand‐searching of cited and citing references produced a further five. Of these 15 articles, incorporating 20 different datasets met the aforementioned criteria. Data were extracted independently and cross‐checked by both authors.

The data are summarised in Table 3, which in column 3 records the specific childhood event being suggested and additional relevant experimental conditions. Column 4 indicates whether there was a significant false feedback effect in comparison with the control condition. Tests purely on within‐group changes in belief before and after the manipulation are not reported, as these may be attributable to regression to the mean or other artefacts of repeated testing. The next two columns quantify the effects obtained by reporting the mean increase on the 8‐point scale typically used and whether the mean increase after false feedback exceeded the scale midpoint. The final column reports the percentage of participants who described themselves as remembering or, in the absence of an actual memory, believing that the suggested event had occurred.

### Results and discussion

Once again, the studies are relatively homogeneous in that most participants imagine events that occurred to them before the age of 10 years. Visual inspection in Table 3 of the mean increased belief ratings before and after false feedback indicates a very consistent advantage for this condition over the control condition. However, like in the imagination inflation studies, analyses of ‘Believers’ indicate that it was usually the minority of study participants who showed clear evidence of responding positively to the intervention (range 18%, in Bernstein et al., 2005a, Study 1, to 53%, in Laney, Morris, Bernstein, Wakefield, & Loftus, 2008, Study 2).

The mean advantage for the false feedback over the no false feedback condition is confirmed by the reported statistics, shown in Table 3, column 4. In the 15 datasets with between‐group tests on mean confidence ratings, 13 found that these differences were significant, one obtained mixed results, and one found no significant differences. As shown in Table 3, column 5, of the 19 datasets where the means or mean change in belief are reported, four involve a maximum increase of less than 1 scale point with false feedback, seven involve a maximum increase of 1–2 scale points and eight a maximum of more than 2 scale points. Only one study reported a belief increase beyond the scale midpoint. Six of the eight datasets reporting these larger increases used guided imagery in addition to false feedback, whereas only one of the 11 datasets reporting smaller increases did so (Clifasefi, Bernstein, Mantonakis, & Loftus, 2013). There is also some indication that the target of the false feedback may play a role in determining the size of the effect. For example, participants' belief ratings actually decreased after false feedback that they had been sick after eating chocolate chip cookies, whereas three of the largest confidence increases came after false feedback concerning attitudes to asparagus.

Studying the subset of participants (‘Believers’) who appear to succumb to the false feedback provides an estimate of the maximum effect that can be achieved. Typically ‘Believers’ reported an increase on the autobiographical belief scale that ranged from 1 to 2 points, remaining below the scale midpoint, for an event at a theme park (Berkowitz, Laney, Morris, Garry, & Loftus, 2008) to 3–5 points, increasing beyond the scale midpoint, for food‐related events (D. M. Bernstein et al., 2005a, 2005b; Laney et al., 2008; Laney et al., 2008; Scoboria, Mazzoni, Jarry, & Bernstein, 2012). In some cases, belief ratings recorded by ‘Believers’ after false feedback were as high as 6.48 on the 8‐point scale (Laney et al., 2008).

A number of false feedback studies also attempted to assess the extent to which participants ‘remembered’ the suggested events rather than just increased their belief they were likely to have happened. Some included ‘memory’ scales as well as ‘belief’ scales at pre‐test and post‐test (Table 1): Of these, two datasets indicated a significant increase with false feedback (Scoboria et al., 2012; Sharman & Calacouris, 2010) and two did not (Scoboria, Lynn, Hessen, & Fisico, 2007, Studies 1 and 2).

Alternatively, investigators have assessed recollective experience at post‐test only. In an early study (Mazzoni, Loftus, Seitz, & Lynn, 1999), ‘some evidence of having a memory’ was found in 44% of the false feedback group, but the majority of the memories presented were clearly speculations or outside the required age range. A number of subsequent studies had their participants choose between indicating that they had a memory of a target event, that they believed the event had occurred but had no memory of it or that they did not believe the event had occurred. These data, shown in Table 3, reveal a wide range of different outcomes: Across five datasets, the proportion of participants ‘remembering’ ranged from 2% to 30%. All the datasets with higher rates of ‘remembering’ involved experiences with foods (Laney et al., 2008, Studies 1 and 2; Laney et al., 2008; Scoboria et al., 2012, 2012).

Of the studies identifying a subset of ‘Believers’, the proportion endorsing a ‘memory’ as opposed to a ‘belief’ fell between 0% and 20% in studies suggesting the participant had been ill after eating something (D. M. Bernstein et al., 2005a, 2005b) or had had a specific experience at a theme park (Berkowitz et al., 2008). This proportion reached 40% in a study suggesting being sick after drinking alcohol (Clifasefi et al., 2013) and 20–50% in studies involving like or dislike of asparagus (Laney et al., 2008; Laney et al., 2008). A study that provided personalised false feedback suggesting that the participant had been sick on peach yoghourt reported that 20% of this group ‘remembered’ the event with their mean scores on a recollective experience scale reaching 6.82 out of 8 (Scoboria et al., 2012).

False feedback studies have shown consistently that participants' autobiographical beliefs can be manipulated under certain experimental conditions, obtaining somewhat stronger average effects than the imagination inflation studies. This appears to be a robust effect found across a wide variety of suggested events. The pattern is similar to the imagination inflation studies in that the majority of participants do not increase their confidence ratings.

Again consistent with the source memory framework (Johnson, 2006), larger increases in confidence are found for events that are more likely to correspond to real‐life experiences. For example, a minority of individuals do respond strongly to false feedback concerning experiences of eating certain foods, particularly if they are novel. It has been suggested that false feedback concerning having been sick as a child may be much more likely to be accepted if the food is associated with unpleasant odours (such as egg salad) or is sour (like yoghourt) (Pezdek & Freyd, 2009). They propose that such effects are likely to be restricted to less appealing foods that are less often consumed. While it is true that the increases in confidence about having been sick on chocolate chip cookies or strawberry ice cream were much lower than after egg salad, suggestions about both liking and hating asparagus led to substantial changes in confidence. The implication is that individuals have complex and differentiated attitudes towards different food types that permit a variety of opportunities for suggestion to alter memory.

As in the imagination inflation studies, increased levels of confidence rarely exceeded the scale midpoint, so that the results might be regarded as describing a reduction in disbelief rather than a positive belief in the event's occurrence. The one exception was the study by Sharman and Scoboria (2009). Rather than the distinctive events used in the original LEI, their version consisted of less specific but highly plausible achievement and affiliation events such as ‘You encouraged a friend or sibling’ and ‘You worked very carefully on an important project’ (Stefanie J. Sharman, personal communication 8 February 2015). A large increase in belief even in the absence of false feedback suggests that, as in Sharman and Barnier's (2008) imagination inflation study, the nature of these events may have been more effective in triggering actual memories.

However, the false feedback research has also involved memory for events prior to age 3 years (Mazzoni & Loftus, 1998; Mazzoni et al., 1999), memory for interactions at Disneyland (Berkowitz et al., 2008) and memory for bone screening (Scoboria et al., 2007). Thus, although for many of the studies, it is difficult to rule out some contribution from actual experience, the range of events studied makes this implausible as an explanation for all the effects obtained.

In comparison with the imagination inflation studies, there has been greater interest in assessing recollective experience as opposed to belief. Rates have been highly inconsistent across studies, and even among ‘Believers’, the range reporting any recollective experience has varied from 0% to 50%. Investigators have relied primarily on self‐report, for example, requiring participants to decide whether their experience could best be described as a memory or a belief. This way of structuring the question does not address issues of memory confidence. The examples provided by Mazzoni et al. (1999) illustrate the wide range of likely ‘memories’ participants in this paradigm may produce. Participants have sometimes been required to write down what they remembered, and it would be valuable to conduct analyses of these more detailed reports, or classify them according to their appropriateness, clarity or completeness. Whereas effects on autobiographical belief are well established, it is probably premature to generalise about the effects of false feedback on ‘full’ memories and important to note that such recollective experiences as are produced may correspond in part to actual experiences rather than to ones that never happened.

## Memory Implantation

The final group of studies, involving the paradigm with the strongest explicit suggestion, has attempted to ‘implant’ a memory of a childhood autobiographical event from scratch, by providing explicit ‘corroboration’ from an authoritative source that the event happened. In a typical design, a parent of the experimental participant is contacted to confirm or disconfirm whether their child experienced a number of specified childhood events. The experimenter usually targets a particular event as the false event (e.g. being lost in a shopping mall or a ride in a hot air balloon), and if the parent indicates it did not happen, the participant is included in the study. Subsequently, participants are encouraged to recall over two or three sessions the details of the false event they are misleadingly told the parent has confirmed as happening. In some cases, they may be shown a doctored photograph that supposedly illustrates their presence at the false event. Unlike the imagination inflation and false feedback paradigms, no systematic baseline measures of belief are taken, and the focus is predominantly on the nature of any recollection. After and during the recall attempts, participants are instructed to provide accounts of the false event and sometimes of comparison true events that have actually been confirmed by the parent. In some studies, as in the other paradigms, they are also guided to imagine the false events as if they had happened. These accounts are then rated for their correspondence to a complete memory, by the investigators as well as sometimes by the participants themselves.

### Method

To be included, a study had to contain a procedure (i) designed to produce a memory of a childhood autobiographical event that on another's authority had not occurred and (ii) that employed ‘corroboration’ specific to the individual participant (such as confirmation by their own family members that the event had happened). From the previously conducted search on Web of Science, Current Contents and MEDLINE, using the joint topic search terms ‘false memor*’ and ‘childhood’, reported in False Feedback Section, 19 articles met the inclusion criteria. Additional hand‐searching of cited and citing references produced a further two articles. After omitting one study in which the target event employed by the researchers contained both true and false segments (Žeželj, Pajić, Omanović, Ninković, & Grčić, 2009), a total of 20 articles incorporating 22 different datasets resulted. Data were extracted independently and cross‐checked by both authors.

The data are summarised in Table 4, which in column 3 details the nature of false events suggested and any relevant between‐subjects conditions. Column 4 indicates if the procedures involved instructions to imagine the false event or not, and the data are presented in columns 5–8. As previously discussed in A Framework for Discriminating Truth and Falsity in One's Own Autobiographical Memories Section, and detailed in Table 1, there have been several investigator‐based rating systems for categorising participants' recollective experiences. Which of the data columns in Table 4 is populated depends on the particular system used. Column 5 reports the percentage of participants describing any recollective experience associated with the false events, including images, memories that were vague, uncertain or partial and speculations. Column 6 reports the percentage of participants describing stronger false recollective experiences that still did not reach our criteria for a ‘full’ memory of requiring evidence for confidence in the recollective experience. These include any recollective experiences left after images not corresponding to memories were explicitly excluded, or experiences where autobiographical belief was measured and incorporated into the false memory rating but confidence in memory was not. Column 7 reports the percentage of participants describing clear memories of false recollective experiences after both images not corresponding to memories and other forms of partial memory were explicitly excluded by the rating system and where there was evidence of confidence in the memory. The final data column gives the means of any self‐report measures of memory for the false event. Included are a measure of autobiographical belief (‘belief in event’), measures that attempt to distinguish whether the participant has a belief or a memory (‘know‐remember’, ‘memory/belief form’), a measure assessing recollective experience (‘memory extent’) and a measure assessing confidence in the recollective experience (‘memory confidence’).

**Table 4:** Studies implanting ‘Corroborated’ false childhood memories of entirely new events in adults

Authors	Participants	False childhood event (additional relevant B/S conditions)	Imagery instruction	% with any false recollective experience^a^	% with false memories partially meeting full criteria	% with full false memories	Mean self‐report recollection/belief ratings
Loftus and Pickrell (1995)	24 adults (age 18–53 years)	Lost in mall	No	25%			
Hyman et al. (1995) Study 1	20 college students	1 selected from birthday with pizza/clown; hospital overnight stay	No	20%			
Hyman et al. (1995) Study 2	51 college students	1 selected from spilt punchbowl at wedding; fire sprinkler in shop; left in car	No	26%		12%	
Hyman and Pentland (1996)	65 college students	spilt punchbowl at wedding (think about event versus guided imagery)	Yes (1 condition)	12% think 36% imagine		9% think 25% imagine	*Memory confidence* (1–7) range^b^ 4.00–3.00 some recall 1.32–1.10 no recall
Pezdek et al. (1997) Study 1	51 college students	Religious event (plausible versus implausible event)	No		6% implausible 22% plausible		
Pezdek et al. (1997) Study 2	20 teens and young adults	Lost in mall; received enema	No		0% enema 15% mall		
Hyman and Billings (1998)	66 college students	Spilt punchbowl at wedding	Yes	27%		15%	*Memory confidence* (1–5) 2.60 clear – 2.25 partial – 1.40 no recall
Porter et al. (1999)	77 college students	1 selected from lost; harmed; attacked; serious accident; serious medical procedure	Yes	56%		26%	
Heaps and Nash (2001)	63 college students	1 selected from LEI^c^ items	Yes	37%			
Wade et al. (2002)	20 college students	Hot air balloon ride with doctored photo	Yes	50%		20%	*Belief in event* [1 (0%) – 7 (100%)] 67% clear – 25% partial – 0% no recall
Lindsay et al. (2004)	45 college students	Put Slime in teacher's desk (sight versus no sight of school class photo)	Yes	46% no photo	23% no photo		*Memory extent* (1–7) 1.91 no photo 2.83 photo *Belief in event* (1–7) 2.36 no photo 3.59 photo
				78% photo	65% photo		
Garry and Wade (2005)	44 young adults	Hot air balloon ride (false narrative versus doctored photo)	Yes	50% photo 82% narrative	29% photo^d^ 41% narrative^d^		*Know – remember* (1–7) 1.36 photo 1.64 narrative *Memory confidence* (1–7) 2.14 photo 2.32 narrative
Ost et al. (2005)	31 college students	1 selected from hospital; lost; eventful holiday or birthday; wedding; contest; serious accident to other	No	23%		3%	*Memory confidence* (1–7) 2.87
French et al. (2006)	58 young adults	Hot air balloon ride False narratives /doctored photos alternated	No	24%	5%		
Desjardins and Scoboria (2007)	44 college students	Put Slime in teacher's desk (presence versus absence of (i) specific and (ii) self‐relevant details in false memory narrative)	Yes	68% self‐relevant 36% no self‐relevant	30% range^b^, ^d^ 55–9%		*Memory extent* (1–8) range^b^ 2.82–1.27 *Belief in event* (1–8) range^b^ 5.18–3.36
Qin et al. (2008) Study 1	33 college students	1 selected from birthday at McDonalds; hospital for injury	Yes (1 condition)	26% range^b^, ^e^ 36–16%		10% range^b^, ^e^ 14–7%	
	86 adults	(Think about versus visualise event; false memory warning versus no warning)					
Strange et al. (2008)	105 young adults	Hot air balloon ride (event at age 2 years versus age 10 years)	No	38% age 2 years 19% age 10 years	13% age 2 years 7% age 10 years		*Know – remember* (1–7) 2.10 age 2 years, 2.20 age 10 years
Wade et al. (2010)	53 young adults	Hot air balloon ride with doctored photo (Photo first versus narrative first)	Yes	41% photo first^d^	10% photo first^d^		
				67% narrative first^d^	23% narrative first^d^		
Short and Bodner (2011)	34 college students	1 selected from ‘plausible’ false event that was another subject's true event	Yes	41%		21%	
Hessen‐Kayfitz and Scoboria (2012)	82 college students	Hot air balloon ride with doctored photo (presence versus absence of (i) self‐relevant and (ii) familiar details in photo)	Yes	34% range^b^ 47–24%	13% range^b^ 19–5%		*Know – remember* (1–7) range 1.74–1.15 *Memory confidence* (1–7) range^b^ 2.17–1.10
Otgaar et al. (2013) Study 1	89 college students	Hot air balloon ride	Yes		36%		(% of participants) *Recollection* 20% *Belief* 20%
Shaw and Porter (2015)	60 college students	1 selected from 3 criminal acts or 3 non‐criminal events	Yes	93%	73% range^b^ 77–70%		*Memory confidence* (1–7) range^b^ 2.86–1

Cells left blank where relevant data not reported.

a Potentially includes clear memories, partial/vague/uncertain memories and speculations, images without memories.

b Means or percentages vary according to condition.

c LEI = Life Events Inventory, Garry et al., 1996.

d Estimated from published figures.

e Author communication.

### Results and discussion

Once again, there is a fairly homogeneous set of studies with participants mainly recalling events supposed to have happened before the age of 10 years, but in one case recalling events from ages 11–14 years (Shaw & Porter, 2015). The percentage of participants reporting any recollective experience (Table 4, column 5) associated with the suggested false event, no matter how vague, uncertain or speculative, ranged from 12% (Hyman & Pentland, 1996, spilt punchbowl at wedding, ‘think’ condition) to 93% (Shaw & Porter, 2015, committed a criminal act). Rates for studies including plausible and implausible events (Pezdek et al., 1997) were averaged across both sorts of event. The weighted average was 47.00% (median 34%). Rates within individual studies varied considerably according to the specific intervention employed and conditions tested. Thus, excluding the two studies that did not employ imagery across all conditions (Hyman & Pentland, 1996; Qin, Ogle, & Goodman, 2008), the weighted mean percentage was 46.3% for the 12 studies using guided imagery but 26.8% for the eight studies not using imagery (Mann–Whitney test *z* = 3.17, *p* < .01). Providing an actual school photo increased the percentage of participants ‘recalling’ putting a manufactured compound called Slime in a teacher's desk from 46% to 78% (Lindsay et al., 2004). Using a narrative description along with a doctored photo of a hot air balloon ride, and presenting the narrative first, boosted recollective experiences considerably (Wade, Garry, Nash, & Harper, 2010).

Applying a more stringent definition of recollective experience, by excluding images not experienced as memories (Column 6), the percentage of participants who ‘recalled’ ranged from 0% for events selected for being implausible (Pezdek et al., 1997, Study 2, receiving an enema) to 65% (Lindsay et al., 2004, put Slime in teacher's desk). The weighted average was 25.45% (median 16%). The lowest rate of ‘recall’ of an event not selected for being implausible, going on a hot air balloon ride in New Zealand, was 5% (French, Sutherland, & Garry, 2006). In contrast, other studies with the same investigator and implanting a ‘memory’ of the same event in the same country achieved rates as high as 41% (Garry & Wade, 2005). Once again, there was considerable variation within studies, and part of this appeared to be due to the provision of personal, self‐relevant information. Thus, inclusion of details like the name of a participant's teacher or photos of familiar individuals significantly boosted the rate of recollective experience (Desjardins & Scoboria, 2007; Hessen‐Kayfitz & Scoboria, 2012; Lindsay et al., 2004).

Using the most stringent definition of recollection in which there is some evidence for both recollective experience and confidence in memory (Column 7), the percentage of participants who ‘recalled’ ranged from 3% (Ost, Foster, Costall, & Bull, 2005) to 26% (Porter, Yuille, & Lehman, 1999), both studies using a variety of events. The upper rate was considerably reduced relative to the previous column, leading to a lower weighted average (15.35%) but a similar median (12–15%).

The final column of Table 4 documents the self‐report data collected by 11 of the 22 studies. Measures of autobiographical belief indicate that mean confidence that false events had actually happened fell mainly in the lower half of the belief scale. Omitted from Table 4 are data on belief in true events that, when reported, was consistently higher (Desjardins & Scoboria, 2007; Lindsay et al., 2004; Wade et al., 2002). However, belief in a false event was much higher in one study when a more stringent definition of a false memory was used (Wade et al., 2002). Measures attempting to distinguish between belief versus recollective experience reported scores heavily weighted towards belief. In one study, where investigator‐based and self‐ratings of whether or not participants had a memory of a false event could be directly compared (Otgaar et al., 2013), the investigator rating (36%) was almost double the self‐rating (20%).

Finally, average scores on measures of recollective experience and confidence in memory for false events all fell at or below the midpoint of the scales used. Even when clear memories were identified by the investigators, participants' confidence in them was below the scale midpoint (Hyman & Billings, 1998). Omitted from Table 4 are data on recollection of true events that, when reported, was consistently higher (Garry & Wade, 2005; Heaps & Nash, 2001; Hessen‐Kayfitz & Scoboria, 2012; Lindsay et al., 2004; Loftus & Pickrell, 1995; Ost et al., 2005; Pezdek et al., 1997; Porter et al., 1999; Shaw & Porter, 2015; Strange et al., 2008). There were no indications that studies using guided imagery obtained higher self‐report ratings of recollective experience or confidence in memory than studies that did not use guided imagery and in one study where these conditions were directly compared use of imagery led to numerically lower confidence in memory (Hyman & Pentland, 1996).

The unique contribution of the memory implantation studies has been to demonstrate that substantial numbers of college students can be encouraged to have false recollective experiences when they are misled by authoritative sources into believing that certain events happened to them in childhood. It is clear, however, that the results of these studies are highly variable and that a high proportion of these ‘memories’ are speculative, partial or only in the form of images. Many participants harbour doubts about their authenticity, and they are not on average rated as being comparable with memories of true events. Attempts to adopt more stringent definitions of what constitutes a memory reduce the mean percentage of participants who respond to the suggestions from 47% to 15%.

There are a number of reasons to question whether all these 15% would meet the requirements for a ‘full’ memory set out by Brewer (1986). In terms of autobiographical belief, the largest effect obtained was a 67% belief that an event for which the participant reported a ‘clear memory’ had actually occurred (Wade et al., 2002). Other studies found that self‐ratings of having a ‘memory’ were much lower even than the more stringent investigator ratings (Otgaar et al., 2013), and even using stringent criteria recollections identified by investigators were only given average confidence ratings by participants for their veracity (Hyman & Billings, 1998). Similarly, Shaw and Porter (2015) found a marked discrepancy between the ease of inducing experiences rated by investigators as memories and the confidence participants had that they were real.

From their own data, Hyman and colleagues (Hyman et al., 1995; Hyman & Pentland, 1996) have argued that through repeated interviews and the social demands of the experiment, misleading information is likely to be integrated into true event memories. This begs the question of whether additional details, frequently included in the criteria for false memory judgements, reflect true memories of actual experiences similar to, but not quite the same, as those being suggested. Hyman and Billings (1998) in a study using visualisation provide such a memory as the one example of a clear false memory of knocking over a punchbowl at a family friend's wedding (edited here):

Interview 2: …Like the table was a round table with a couple of chairs sitting around it and were under the shade of a tree. They were right there and there were some drinks on the table, yeah, I think there was just drinks on the table, I don't think there was an actual pitcher or anything. I think when I just bumped the table, or maybe I bumped the glasses or something, it kind of just spilled on them, so I think what I did was I bumped the table, I liked bumped the table and the glasses tipped over on them (p. 10).

One hint that the distinction between memory and imagination might not always be easy or possible for judges to make from the false event description comes from our finding that, while guided imagery instructions led to significantly higher rates of investigator‐based false memory judgements, self‐reports of belief in and memory of false suggested events are similarly low for studies with and without guided imagery instructions. This is consistent with the source memory framework (Johnson, 2006), which predicts that the act of imagination may create additional contents that could be confused with real memories, but may also lead the person to discount those contents as being due to the mental operations employed. This suggests that when guided imagery is used, unless participants are explicitly asked about the extent to which their accounts reflect the productions of their imagination based on the experimenter's instructions (as opposed to actual memories), judgements of false memories are at risk of being inflated.

A related issue to the possible miscategorisation of true memories, because of their similarity to false events, is whether some memories deemed false by investigators on the authority of an older family member may have actually happened (Desjardins & Scoboria, 2007; Porter et al., 1999). Extensive reviews of documented childhood experiences have found that people are more likely to have forgotten past negative events they have experienced than to have remembered events they have not (Brewin, Andrews, & Gotlib, 1993; Hardt & Rutter, 2004). These reviews point to evidence that younger and older people's reports of their childhoods are more in accord with those of independent observers than with their parents' reports. The agreement between parents and their children on reports of stressful life events is also weak (Sandberg et al., 1993). One reason for this is that childhood experiences outside of the home may not be known about or shared with parents. All this suggests that events cannot always be assumed to be false on the authority of a parent or older relative, because of them forgetting and sometimes not being in a position to know.

The issue is very relevant to data from studies such as that of Shaw and Porter (2015) that purport to show very high percentages of participants generating false memories of ‘criminal acts’. It is not clear how many of these acts would have been deemed criminal by independent observers because the examples cited in the paper refer to everyday events that are common in the lives of teenagers, such as minor scuffles and fights. But, underscoring the likelihood that these might at least in part be based on real memories, a large representative survey in the UK found that almost half of young people aged 11 to 17 years reported committing at least one criminal act (Beniart, Anderson, Lee, & Utting, 2002).

## General Discussion

With the controversy over child sexual abuse allegations in mind, the main interest of the false memory studies has been the extent to which individuals can come to accept the reality of childhood events that never happened. In the absence of a consistent approach to these questions, and attempting to account both for the variability in the empirical data and the acknowledged complexity of the judgements involved, we applied a framework derived from Brewer (1986) that discriminates different types of autobiographical memory experience. In this final section, we summarise the conclusions suggested by the research findings.

The imagination inflation studies were by and large only intended to assess autobiographical belief, so that the extent to which these procedures produce full autobiographical memories is unknown. However, reports of very large increases in belief following imagination inflation might indicate the creation of a full false memory. In the one study reporting this information, this effect occurred for approximately 5% of imagined items (Garry et al., 1996). Such information concerning individual variability is highly desirable, as mean differences do not identify how many participants respond strongly to the experimental procedures. We would strongly encourage the routine reporting of these data in the future.

The false feedback studies have gone to greater lengths to assess recollective experience but achieved inconsistent results, even when the same false event was suggested. Measures have largely relied on a self‐report and on participants' understanding of the difference between belief and memory, but with little systematic follow‐up to confirm the validity of their responses. Given the known difficulty participants have in interpreting questions about memory (Ost et al., 2008; Sjöden, Granhag, Ost, & Roos af Hjelmsäter, 2009; Smeets et al., 2006; Smeets et al., 2009), it would probably be premature to estimate the number of full autobiographical memories and to distinguish these from actual experiences, for example, of eating asparagus or yoghourt. It is interesting that despite these uncertainties, a number of the studies succeeded in bringing about small behavioural changes in their participants, for example, changing the amount of available food consumed in the laboratory (Scoboria, Mazzoni, & Jarry, 2008; Scoboria et al., 2012).

Where larger effects were obtained, for example, among ‘Believers’ who responded more strongly to the false feedback manipulations, they were mainly limited to the suggestion of common experiences, for example, involving certain sorts of food, or less distinctive experiences that as well as being common were more open to interpretation (Sharman & Calacouris, 2010). These results are consistent with the source memory framework that predicts that importing features of real experiences decreases the accessibility of disconfirming information and makes it more likely that suggested false events are accepted. The greater impact of the deceptive information provided in the false feedback studies, over imagination alone, is consistent with research on persuasive communications that the degree of change in attitudes and opinions is greater, the more credible or trustworthy the source of information (Pornpitakpan, 2004).

The memory implantation studies have gone to greater lengths to deceive participants and have largely relied on sources of evidence, such as parents, that fulfil all the criteria for establishing trust in communication: expertness relevant to the topic under discussion, reliability as an information source and the possession of favourable intentions towards the recipient (Giffin, 1967). Although these methods, particularly when combined with the use of guided imagery, have produced substantial quantities of recollective experiences, their ability to produce full autobiographical memories is more equivocal. From our review of studies using more stringent definitions of recollection, the upper bound would seem to be about 15% of participants, but for reasons reviewed previously, particularly the discrepancies between observer‐ratings and self‐ratings of memory experiences, the actual figure may be lower.

These data are inconsistent with claims (e.g. Conway, 2013) that it is easy to create false memories of childhood in others. Our review indicates that the majority of participants are resistant to the suggestions they are given, despite repeated attempts to remember, the use of guided imagery, doctored photos, the involvement of trusted others and what Hyman and Pentland (1996) noted were the very high levels of demand exercised on college students. Also, it has become apparent from the false memory examples published that those who do have recollective experiences are often cautious and uncertain about them. The data strongly support the source memory perspective that emphasises the complex judgements involved in evaluating truth and falsity in autobiographical memory. Findings that participants also discriminate appropriately in their ratings of clarity, confidence, and so on between true and false memories, and produce more details for true than false events (Qin et al., 2008), also testify to the appropriate operation of source memory judgements.

Even if the numbers of full false memories created by these procedures are few, it is still necessary to explain how some people plainly are convinced that these events have occurred. In this respect, it is worth quoting at some length the observations of Ira Hyman, a pioneer in the field (Hyman & Pentland, 1996):

In all of our studies, the suggestions regarding an event do not appear to be adopted wholesale. Rather what appears to occur is that the individual considers the suggestions in light of other self‐knowledge (self‐schema, personal memories, etc.), and constructs a memory that is a combination of the suggestion plus related self‐knowledge. Hyman et al. (1995) and Hyman and Billings (1995) both reported that individuals who talked about related self‐knowledge in the early interviews were more likely to create a false memory by the end of the experiment (p. 112).

Consistent with this, several studies have demonstrated that the inclusion within the experimental procedure of personal, self‐relevant details like photographs of familiar individuals boosts recollective experience (Desjardins & Scoboria, 2007; Hessen‐Kayfitz & Scoboria, 2012; Lindsay et al., 2004). It is plausible that these details act as cues, aiding the retrieval of genuine self‐knowledge and therefore the subsequent construction of false memories in the way described by Hyman.

The possibility raised by these researchers that even full false memories are at least partially constructed from veridical autobiographical elements, and the independence of measures of recollective experience from autobiographical belief (Scoboria et al., 2014), has important implications for future research. They suggest that there should be a more formal way of establishing the existence of a personal memory that takes into account the existence of a recollective experience together with confidence in that memory. One way of achieving this as suggested by Ost, Scoboria, Smeets and others (Ost et al., 2008; Sjöden et al., 2009; Smeets et al., 2006; Smeets et al., 2009) is that memory reports are followed up with individual questioning designed to have participants detail their confidence in different elements of their ‘memory’ and to make corresponding source judgements. It would also be valuable to test whether the increased false memory rate produced by personally relevant details is due to the retrieval of additional veridical elements that then find their way into recall of the suggested events or to the retrieval of a true event similar to the one that is being suggested.

Given the discrepancies between investigator and self‐report judgements of false memories, future research could also address how experimenters assess the content of false memory descriptions when guided imagery is included in instructions to remember. In studies using this intervention, participants, with no prior memory of the false event, are asked to imagine details as if the event had actually happened, such as what it might have been like, what was seen, who was present, and what the weather was like. The question therefore arises whether experimenters incorporate accounts of such imaginings into the content of the event description used to determine the existence of false memories. Although the majority of studies distinguish images without memories from actual memories in their rates of false memories, most studies do not make clear exactly how this is performed.

### Conclusions and implications for memories of child sexual abuse

Authors in the majority of the studies reviewed previously have claimed that their research questions and data are relevant to the controversy concerning false memories of childhood trauma, particularly child sexual abuse, being created by therapists. It is therefore important to consider how their procedures might be mirrored in the real world, how the different approaches to measurement should be described and what might be appropriately concluded from this body of work for presentation in a court of law.

One issue is the extent to which the experiments create conditions that are similar to those that might occur in a course of therapy. Thus, some therapists might plausibly have patients try to recall by vividly imagining hypothetical events, guiding them with specific questions and do this repeatedly, as in the imagination inflation studies. They might provide plausibility information, for example, concerning the high proportion of their patients who had been sexually abused, or state that their patients' symptoms made it likely that they had been abused, as in the false feedback studies. They would not be likely to provide more elaborate forms of corroboration, such as the doctored photos or parental statements that feature in the memory implantation studies.

Second, the plausibility of events suggested by therapists depends crucially on the individuals' knowledge of the setting in which it may have occurred and the other persons who may be involved. Despite the fact that the false memory studies have not yet demonstrated, it is possible to implant memories for events involving intimate transactions with family and close others, an upbringing involving exposure to chaotic, neglectful or sexually inappropriate others may provide a background in which such suggestions are more likely to be regarded as plausible and accepted. More problematic are memories for abuse that, as is often the case, is repeated, sometimes with very great frequency. A challenge for the future will be to demonstrate that it is possible to implant memories of a repeated event. For the present, this should be noted as an important limitation in court reports.

Thirdly, it should be noted that as sources of information therapists enjoy several advantages. If a successful therapeutic relationship has been established, they are likely to be trusted. High levels of trust would coincide with perceived expertise, making their persuasive communications more likely to be accepted (Giffin, 1967). They are also in a position to draw attention to, or encourage patients to retrieve, very specific personally relevant details that might increase the amount of recollective experience around suggested events. Unlike parents, however, they are not in a position to make statements from personal experience about what did or did not happen during their patients' childhoods, thus reducing their informational value as sources for suggested memories.

In referring to this literature in courtroom testimony, it will be important to be explicit about the type of effects being described (e.g. do they involve ‘full’ or ‘clear’ memories), and the size of any effects obtained (e.g. do they indicate a belief that an event was more likely to have happened than not). It should be noted that in these experiments, repeated attempts to remember have been shown to lead to the recovery of true autobiographical memories, as well as to false memories containing true elements (Hyman et al., 1995; Hyman & Pentland, 1996). Similarly, clinicians have argued that in their practice, false memories tend not to arise *de novo* but are often based in part on memories of real events (Mollon, 1998). Other limitations need to be mentioned. For example, practical and ethical constraints mean it is difficult to assess the durability of false memories over the periods of time typical of legal cases. Nor is there yet evidence to show that false memories can be created with the degree of conviction necessary to sustain protracted legal proceedings involving the police and cross‐examination in the courts. On the other hand, therapists may have been able to wield considerably more suggestive influence than was possible in these brief laboratory studies.

As regards the terminology used in court reports, it is clear that use of the term ‘false memory’ without qualification or explanation has the potential to be misleading. At minimum, we recommend that experts wanting to refer to this literature need to educate courts about the conditions needed to establish the presence of an autobiographical memory, and to draw attention to the difference between autobiographical belief, recollection, and confidence in memory when discussing the results of these studies. It will also be important to describe the age of the participants and the nature of the events that have been suggested or falsely recalled in these studies and not to generalise from studies of food preference, say, to more distinctive events.

In summary, there seem to us to be a number of conclusions that can and cannot be properly drawn from the existing experimental literature. On the one hand, it has provided a valuable demonstration that compelling false memories can sometimes be created even with the restrictions imposed by laboratory research. There are sufficient grounds to conclude that a (probably small) minority of people might develop false memories of childhood events with these characteristics and that any such memories might contain a mixture of true and false elements. The fact that susceptibility to false memories appears to be lower than has often previously been suggested does not diminish in any way the significant implications for the courtroom and the need to consider the extremely damaging consequences that might ensue from sincerely believed but false accusations. On the other hand, we believe it cannot be concluded that false memories of childhood events possessing these characteristics are common, that they are easy to suggest or implant or that the majority of individuals are susceptible to them.

The literature we have reviewed provides valuable guidelines as to the conditions under which such false memories are more likely. The situations of most concern are those in which the following occur repeatedly and simultaneously: (i) autobiographical belief is strengthened by plausibility information supplied by a trusted figure; (ii) recollective experience is increased by encouraging and guiding imagery, particularly when it is prompted by personally relevant details; and (iii) confidence in the veracity of the resulting experiences is boosted by an uncritical acceptance of them. An example would be that of therapists who attribute their patients' symptoms to child sexual abuse in the absence of any explicit memory of it, instruct their patients in how to imagine this scenario and accept uncritically any material produced by this procedure. It should be emphasised, however, that false memories do not necessarily require any external influence and may sometimes arise from the spontaneous misinterpretation of internal dreams or images (Brewin, Huntley, & Whalley, 2012; Rassin, Merckelbach, & Spaan, 2001).

Nevertheless, it is of great concern that, unlike the great majority of qualified clinical psychologists, some alternative therapists continue to endorse mistaken ideas, for example, that people's conscious memories can go back to birth and that hypnotically retrieved memories are reliable (Andrews et al., 1999; Brewin & Andrews, 2014). These beliefs may foster the inappropriate practices we and others have identified. Given the generally accepted view by professional bodies and independent commentators that recovered memories may be genuine, false or a mixture of the two (Lindsay & Read, 1995; Wright, Ost, & French, 2006), expert witnesses are in a good position to evaluate the extent to which the conditions conducive to false memories have or have not been present prior to memory recovery. The research on creating false childhood memories has been extremely helpful in illuminating these processes and constitutes an important part of the knowledge base that experts testifying in historic abuse cases must consider.
